# Assessing the validity of a data driven segmentation approach: A 4 year longitudinal study of healthcare utilization and mortality

**DOI:** 10.1371/journal.pone.0195243

**Published:** 2018-04-05

**Authors:** Lian Leng Low, Shi Yan, Yu Heng Kwan, Chuen Seng Tan, Julian Thumboo

**Affiliations:** 1 Department of Family Medicine & Continuing Care, Singapore General Hospital, Singapore, Singapore; 2 Family Medicine, Duke-NUS Medical School, Singapore, Singapore; 3 SingHealth Regional Health System, Singapore Health Services, Singapore, Singapore; 4 Duke–NUS Medical School, Singapore, Singapore; 5 Singapore Heart Foundation, Singapore, Singapore; 6 Saw Swee Hock School of Public Health, National University of Singapore, Singapore, Singapore; 7 Office of Insights and Analytics, SingHealth, Singapore, Singapore; 8 Health Services Research Centre, Singapore Health Services, Singapore, Singapore; 9 SingHealth Regional Health System, Singapore Health Services, Singapore, Singapore; 10 Department of Rheumatology and Immunology, Singapore General Hospital, Singapore, Singapore; Yokohama City University, JAPAN

## Abstract

**Background:**

Segmentation of heterogeneous patient populations into parsimonious and relatively homogenous groups with similar healthcare needs can facilitate healthcare resource planning and development of effective integrated healthcare interventions for each segment. We aimed to apply a data-driven, healthcare utilization-based clustering analysis to segment a regional health system patient population and validate its discriminative ability on 4-year longitudinal healthcare utilization and mortality data.

**Methods:**

We extracted data from the Singapore Health Services Electronic Health Intelligence System, an electronic medical record database that included healthcare utilization (inpatient admissions, specialist outpatient clinic visits, emergency department visits, and primary care clinic visits), mortality, diseases, and demographics for all adult Singapore residents who resided in and had a healthcare encounter with our regional health system in 2012. Hierarchical clustering analysis (Ward’s linkage) and K-means cluster analysis using age and healthcare utilization data in 2012 were applied to segment the selected population. These segments were compared using their demographics (other than age) and morbidities in 2012, and longitudinal healthcare utilization and mortality from 2013–2016.

**Results:**

Among 146,999 subjects, five distinct patient segments “Young, healthy”; “Middle age, healthy”; “Stable, chronic disease”; “Complicated chronic disease” and “Frequent admitters” were identified. Healthcare utilization patterns in 2012, morbidity patterns and demographics differed significantly across all segments. The “Frequent admitters” segment had the smallest number of patients (1.79% of the population) but consumed 69% of inpatient admissions, 77% of specialist outpatient visits, 54% of emergency department visits, and 23% of primary care clinic visits in 2012. 11.5% and 31.2% of this segment has end stage renal failure and malignancy respectively. The validity of cluster-analysis derived segments is supported by discriminative ability for longitudinal healthcare utilization and mortality from 2013–2016. Incident rate ratios for healthcare utilization and Cox hazards ratio for mortality increased as patient segments increased in complexity. Patients in the “Frequent admitters” segment accounted for a disproportionate healthcare utilization and 8.16 times higher mortality rate.

**Conclusion:**

Our data-driven clustering analysis on a general patient population in Singapore identified five patient segments with distinct longitudinal healthcare utilization patterns and mortality risk to provide an evidence-based segmentation of a regional health system’s healthcare needs.

## Introduction

The aging population with increasing chronic diseases burden is a global challenge that has resulted in escalating healthcare expenditure and strained healthcare resources [1]^,^[2]. Recently, there has been a growing interest in population health management among integrated health systems worldwide to understand the determinants of health. This drives intervention priorities to improve the overall health outcome of an entire population with more efficient healthcare delivery at better value [3]^,^[4]. To achieve these goals, it is imperative to have a deep understanding of the heterogeneous health status and specific healthcare needs of different groups of patients served by a healthcare system and match healthcare services to each group of patients [5]. This patient-centered approach in delivering healthcare requires health services to be organized around segments of patients with similar healthcare needs across care continuum, regardless of their individual diseases status [6]^,^[7]^,^[8]. Segmentation of a heterogeneous population into parsimonious and relatively homogenous groups with similar healthcare needs can enable healthcare policymakers to optimize healthcare service planning at population level and develop integrated healthcare system that is more targeted and efficient [9]^,^[10]^,^[11].

Two major approaches for population segmentation have emerged over the years. Expert-driven approaches define segments a-priori by experts’ consensus, while data-driven approaches employ post-hoc statistical analysis such as clustering analysis or latent class analysis on empirical data to segment a population. Examples of expert-driven approaches include the Johns Hopkins Adjusted Clinical Group (ACG) System [12] and the Clinical Risk Group (CRG) system by 3M [13]. Both systems utilize diagnostic codes as input data to classify patients into one of over 200 mutually exclusive risk groups. Patients are segmented into a mutually exclusive category based on a hierarchical system of classification where greater weightage is given to patients’ highest morbidity diseases. Both segmentation systems have been validated through association with differing healthcare utilization associated costs. However, both proprietary tools require a comprehensive electronic medical record system and the health system to utilize similar set of disease codes in order to function optimally. Another example of expert driven approaches is the Senior Segmentation Algorithm developed by Kaiser Permanente for elderly persons (“robust seniors without chronic conditions”, “seniors with one or more chronic conditions”, “seniors with advanced illness and end-organ failure”, “seniors with advanced frailty or at the end of life”) [14]. The Bridges to Health model is a conceptual segmentation framework that characterizes population segments by health priorities, while Delaware and North West London input demographic information such as age, disease condition information based on diagnostic codes and functional ability into its segmentation algorithm logic [15]^,^[16]^,^[17]. The Complexdex segmentation system utilizes patient physical and mental diagnosis as well as lifestyle relevant information as inputs to determine patient segments [18]. As expected, there is no widely accepted consensus or generalizable expert-defined criteria on the optimal number of segments and the definition of each segment for different populations.

Data-driven approaches provide an attractive alternative, generating evidence-based insights of a population’s health status from large volumes of patient healthcare data to support policy decisions on population health[19]. Liu et al’s study of the Taiwan National Health Insurance Survey participants examined detailed demographic, medical, socio-economic data and clustered the population into four groups: “Relative Healthy”, “High Comorbidity”, “Functional Impairment”, and “Frail” [20]. These four groups were further shown to have significantly different healthcare needs [21]. Van der Laan et al’s (demand-driven segmentation model) and Lafortune’s latent class analysis of SIPA trial examined self-reported biological, psychological, functional and social domain inputs and demonstrated the predictive ability of the segments for healthcare service utilization [22]^,^[23].

Traditionally, demographics such as age and disease variables including symptoms severity, duration of diseases are commonly used criteria for data-driven population segmentation [22]^,^[24]^,^[25]^,^[26]. However, these variables provide an indication of but may not necessarily reflect actual healthcare needs across care continuum[19]. For example, for those who had the same diagnosis “asthma”, some may only need ambulatory monitoring and management whereas others require intensive care in tertiary centers [24]. Healthcare utilization has been increasingly recognized as a proxy for the health needs of different subgroups within a population[27]. Recently, Zayas et al. used one year cross-sectional data to demonstrate the potential of clustering analysis on healthcare utilization data to uncover meaningful utilization patterns in different patient segments with distinct care priorities [28]. Data on healthcare utilization can provide more quantitative information on healthcare needs that enable policymakers to practice evidence-based healthcare resource planning, further analyze high utilization segments and develop focused health intervention strategies. However, to the best of our knowledge, no study has examined the predictive ability of these data-driven segmentation methodologies for longitudinal follow up healthcare utilization and mortality data.

In this study, we therefore aim to evaluate a data-driven, healthcare utilization-based clustering analysis to segment a general patient population within the largest public healthcare organization in a multi-ethnic Asian city, Singapore. We first assessed whether the clustering analysis is able to generate segments of patients with unique healthcare utilization patterns and disease profiles. Secondly, we examined the validity of our cluster-derived segments on their discriminative properties on 4-year healthcare utilization, mortality and association with clinical chronic diseases.

## Methods

### Study site, data sources

The Singapore Health Services Regional Health System (SingHealth RHS) is the largest RHS in Singapore providing integrated care (tertiary hospitals, community hospitals, and primary care clinics) for a specific geographic region in Singapore. A Regional health system (RHS) population health datamart was constructed to support population health management in SingHealth in 2016 by extracting deidentified clinical, administrative and other data from the SingHealth Data warehouse. These data included demographics, chronic disease status (based on International Classification 9 and 10 codes), public healthcare utilizations including inpatient admissions, specialist outpatient visits, emergency department visits, primary care clinic visits, and mortality from years 2012 to 2016. We included all adult patients (21 years of age and above) who are Singapore residents (Singapore citizens and permanent residents) and utilized services in institutions belonging to the SingHealth in 2012. The chronic diseases were selected based on Singapore Chronic Disease Management Program, Charlson Comorbidity Index [29] and Elixhauser Index [30] (Supplementary Table 1). In order to reflect accurate health utilization patterns, we excluded patients who do not reside in our SingHealth RHS catchment population (defined as population residing in postal codes served by the SingHealth RHS). Approval for data retrieval was obtained from the relevant approving authorities. The SingHealth Centralized Institutional Review Board (CIRB 2016/2294) approved this study for ethics.

### Segmentation variables

Age and healthcare utilization data in year 2012 were used for segmentation. Age is an important health determinant with profound implications on healthcare needs, health related beliefs and behaviors, which are important considerations for health and social services integration [31].

Healthcare utilization data included inpatient admissions, specialist outpatient clinic attendances, and emergency department attendances to all SingHealth hospitals (Singapore General Hospital Campus, Kandang Kerbau Women and Children Hospital). Primary care utilization is recorded for attendances to SingHealth Polyclinics, one of two large polyclinic clusters in Singapore. Polyclinics are large public primary care clinics that provide government subsidized primary care.

### Cluster analysis

Variables were transformed to z-scores using means and standard deviations because scales differed across variables. Clustering analysis by k-means method with a Euclidean distance was chosen because it can generate reproducible cluster solutions with reduced risk of cluster mis-assignment common with hierarchical cluster methods [32]. It has also been used extensively as a clustering technique in epidemiological studies, and can deal with large datasets efficiently [33]^,^[34]. However, k-means requires the desired number of clusters (k) to be pre-determined as input. Hence, hierarchical cluster analysis (Ward’s linkage) was first used to determine k because it maximized the within-group homogeneity [26]. As it struggles with large datasets, a method commonly used in other studies was applied, which was to run hierarchical cluster analysis (Ward’s linkage) on 10 random samples of 3000 patients, for each of which k = 2 through 15 were compared [19]^,^[35]. The selection of k was based on the following criteria: 1) the pseudo F statistic, which assesses the cluster tightness should be low and 2) high Duda/Hart Je(2)/Je(1) index should be high with a corresponding low pseudo T-squared value, with high pseudo T-squared values on either side [36]^,^[37]^,^[38]. The selected k was then used to perform a k-means analysis of the full dataset, which generates non-overlapping population segments. The final segmentation outcome was evaluated by its clinical relevance and interpretability.

### Statistical analysis

We examined whether there are significant differences in demographics, chronic disease patterns and healthcare utilization in 2012 between segments using Chi-square test/Fisher exact test and one-way ANOVA test/Kruskal-Wallis H test with Bonferroni correction for categorical variables and continuous variables (parametric and nonparametric) respectively.

In order to validate the identified segments, we assessed their discriminative properties according to healthcare utilizations and mortality from 2013 to 2016. To determine whether there are significant differences in the public healthcare utilization from year 2013 to 2016, we first excluded those who deceased in 2012 and thus would not have any utilization from 2013 to 2016 (n = 1,284 patients). We began with bivariate analysis between the population segment and the public healthcare utilization (nonparametric) and mortality from year 2013 to 2016 using Kruskal-Wallis H test, and Chi-square test, respectively. For count outcomes (i.e., public healthcare utilization over 4 year period) we used Poisson or negative binomial regression where appropriate to examine the relationship between population segment membership and healthcare utilization 2013–2016. The cluster groupings is the exposure of interest and we have adjusted for age, gender, ethnicity and public healthcare utilization in 2012. In view that there are people who would die, we have included the offset term, which is the log of the follow-up time which starts on 01 Jan 2013 and ends on 31 Dec 2016 (or the death date) for participants who did not experience death before 1 Jan 2017 (or had died between 01 Jan 2013 and 31 Dec 2016). We also conducted pairwise segment comparison using one-degree freedom Chi-square test, and evaluated the overall p-value for 5 cluster groupings with the likelihood ratio test with 4 degrees of freedom. To assess the independent relationship of mortality rate between segments, multivariate Cox proportional hazard regression analysis was performed. Hazard Ratio (HR) and its accompanying 95% confidence interval are also presented. Finally, Kaplan Meier estimator was used to estimate the survival function from lifetime data, and log-rank test to determine significance. Kaplan-Meier survival curves were plotted using the start date of 1st January 2013 as time of entry into the study for all patients since the segment membership for patients were determined using healthcare utilization data accumulated in year 2012. We calculated the time to survival as the number of days from entry to death (for patients who are deceased on or before 31st December 2016) or 1461 days (number of days from entry to 31st December 2016 for censored patients who were not deceased until the end of the study period). Differences in the survival plots were analyzed using log-rank test. All analyses were performed on STATA/SE 14 (TX, USA).

## Results

### Segmentation outcome

Hierarchical clustering analysis (Ward’s linkage) identified the optimal number of clusters k to be 3–8 based on the criteria described above. The k-means cluster analysis was conducted for k = 3–8 and we chose k = 5 as the final desired number of segments based on size of the smallest cluster (at least 1% of sample) and interpretability of the segments [39].

146,999 patients were segmented into five clusters based on clustering variables (age and public healthcare utilization patterns) (Table 1). The five segments were defined and labeled to best represent their health services utilization and chronic disease patterns in the individual clusters. Therefore, we named segments 1 to 5 as “Young, healthy”; “Middle age, healthy”, “Stable, chronic disease”; “Complicated chronic disease”; and “Frequent admitters” respectively.

**Table 1:** Segmentation outcome and characteristics.

	All	Segment 1: Young, healthy	Segment 2: Middle-age, healthy	Segment 3: Stable, chronic disease	Segment 4: Complicated chronic disease	Segment 5: Frequent admitters	p-value
**Number of patients (%)**	146,999 (100%)	41,171 (28.01%)	38,400 (26.12%)	41,184 (28.02%)	23,614 (16.06%)	2,630 (1.79%)	
Age in 2012 (Years, mean (SD))	49.8 (17.2)	28.85 (4.58)	43.99 (4.37)	59.07 (4.51)	76.14 (6.36)	61.06 (11.31)	<0.001
**Healthcare Utilization 2012**							
Inpatient Admissions (Mean (SD))	0.15 (0.58)	0.13 (0.42)	0.08 (0.37)	0.10 (0.49)	0.26 (0.78)	1.27 (1.83)	<0.001
Specialist Outpatient Clinic Visits (Mean (SD))	2.88 (5.96)	2.24 (4.11)	1.99 (3.28)	2.27 (3.41)	3.15 (4.40)	32.78 (17.11)	<0.001
Emergency Department Visits (Mean (SD))	0.19 (0.70)	0.16 (0.63)	0.14 (0.56)	0.16 (0.65)	0.31 (0.89)	0.90 (1.45)	<0.001
Primary Care Clinic Visits (Mean (SD))	2.84 (4.25)	1.60 (2.93)	2.13 (3.21)	3.63 (4.84)	4.42 (5.18)	3.58 (5.96)	<0.001

### Demographics

As shown in Table 1, the overall mean age of the study population was 50.13 years with standard deviation (SD) of 17.3. Subjects in the “Young, healthy” segment are the youngest (mean 28.85, SD 4.58), followed by subjects in the “Middle age, healthy” segment (mean 43.9, SD 15.6). Subjects in the “Complicated chronic disease” segment are the oldest (mean 76.14, SD 6.36). The differences in age between the segments are statistically significant with p < 0.001. The majority of the study population was Chinese, and ethnicity trends was similar to the Singapore population [40].

### Chronic disease patterns

The five segments also had significantly different chronic disease patterns (Table 2) in 2012. The prevalence of stable chronic diseases such as diabetes without complications, hypertension, hyperlipidemia, benign prostatic hypertrophy in this group were lower than population average in the “Young, healthy” and “Middle age, healthy” segments but higher than population average in the “Stable, chronic disease” segment. The “Complicated chronic disease” segment had the highest proportion of patients with chronic stable diseases. The “Frequent Admitters” segment had a high burden of complex chronic diseases and organ failures, including End Stage Renal Failure (1.5%), Chronic Obstructive Pulmonary Disease with Cor Pulmonale (5.6%), Diabetes with complications (4.5%), Atrial fibrillation (5.1%), Chronic Liver Disease (3.3%), Malignancy (31.2%), Heart Failure (7.4%), and Peripheral Vascular Disease (4.5%).

**Table 2:** Demographics and chronic disease patterns by segments.

	All	Segment 1: Young, healthy	Segment 2: Middle-age, healthy	Segment 3: Stable, chronic disease	Segment 4: Complicated chronic disease	Segment 5: Frequent admitters	p-value
Gender (Male %)	62108 (42.3%)	15611 (40.0%)	15829 (41.8%)	18850 (44.6%)	10763 (42.7%)	1055 (40.2%)	<0.001
Ethnicity							<0.001
Chinese (%)	115360 (78.5)	25981 (66.6)	28809 (76.1)	35879 (84.9)	22447 (89.0)	2244 (85.5)	
Malay (%)	11260 (7.7)	4048 (10.4)	3402 (9.0)	2468 (5.8)	1155 (4.6)	187 (7.1)	
Indian (%)	14578 (9.9)	6730 (17.3)	3753 (9.9)	2870 (6.8)	1111 (4.4)	114 (4.3)	
Others (%)	5801 (3.9)	2250 (5.8)	1910 (5.0)	1051 (2.5)	510 (2.0)	80 (3.0)	
**Stable Chronic Diseases**							
Diabetes without chronic complication (%)	20753 (14.1)	443 (1.1)	2505 (6.6)	8826 (20.9)	8308 (32.9)	671 (25.6)	<0.001
Hypertension (%)	42979 (29.2)	773 (2.0)	5188 (13.7)	18029 (42.7)	17661 (70.0)	1328 (50.6)	<0.001
Chronic kidney disease Stages 3 & 4 (%)	4576 (3.1)	72 (0.2)	375 (1.0)	1393 (3.3)	2361 (9.4)	375 (14.3)	<0.001
Asthma (%)	4951 (3.4)	1488 (3.8)	964 (2.5)	1338 (3.2)	1046 (4.1)	115 (4.4)	<0.001
Hyperlipidemia (%)	42373 (28.8)	656 (1.7)	5642 (14.9)	18809 (44.5)	16032 (63.6)	1234 (47.0)	<0.001
Osteoarthritis (%)	16771 (11.4)	757 (1.9)	2899 (7.7)	6996 (16.6)	5524 (21.9)	595 (22.7)	<0.001
Osteoporosis (%)	640 (0.4)	5 (0.0)	10 (0.0)	156 (0.4)	432 (1.7)	37 (1.4)	<0.001
Benign Prostatic Hypertrophy (%)	1031 (0.7)	5 (0.0)	52 (0.1)	368 (0.9)	572 (2.3)	34 (1.3)	<0.001
Chronic Obstructive Pulmonary Disease without cor pulmonale (%)	3074 (2.1)	534 (1.4)	399 (1.1)	703 (1.7)	1286 (5.1)	152 (5.8)	<0.001
Hyperthyroidism (%)	1190 (0.8)	224 (0.6)	364 (1.0)	410 (1.0)	166 (0.7)	26 (1.0)	<0.001
Hypothyroidism (%)	1913 (1.3)	114 (0.3)	422 (1.1)	748 (1.8)	567 (2.2)	62 (2.4)	<0.001
**Complex Chronic Diseases**							
Diabetes with chronic complications (%)	2160 (1.5)	21 (0.1)	180 (0.5)	858 (2.0)	982 (3.9)	119 (4.5)	<0.001
Cerebrovascular disease (%)	5140 (3.5)	46 (0.1)	366 (1.0)	1589 (3.8)	2889 (11.5)	250 (9.5)	<0.001
Chronic Kidney Disease Stage 5 or End Stage Renal Failure (%)	1774 (1.2)	42 (0.1)	125 (0.3)	401 (0.9)	905 (3.6)	301 (11.5)	<0.001
Chronic Obstructive Pulmonary Disease with cor pulmonale (%)	2563 (1.7)	530 (1.4)	375 (1.0)	582 (1.4)	930 (3.7)	146 (5.6)	<0.001
Major depression (%)	2797 (1.9)	678 (1.7)	630 (1.7)	781 (1.8)	607 (2.4)	101 (3.8)	<0.001
Schizophrenia (%)	559 (0.4)	50 (0.1)	158 (0.4)	205 (0.5)	134 (0.5)	12 (0.5)	<0.001
Dementia (%)	505 (0.3)	1 (0.0)	2 (0.0)	22 (0.1)	467 (1.9)	13 (0.5)	<0.001
Bipolar disorder (%)	32 (0.0)	5 (0.0)	10 (0.0)	10 (0.0)	6 (0.0)	1 (0.0)	0.69
Collagen Vascular diseases (%)	516 (0.4)	79 (0.2)	87 (0.2)	126 (0.3)	155 (0.6)	69 (2.6)	<0.001
Anxiety (%)	1290 (0.9)	287 (0.7)	338 (0.9)	397 (0.9)	219 (0.9)	49 (1.9)	<0.001
Parkinson’s disease (%)	475 (0.3)	0 (0.0)	7 (0.0)	94 (0.2)	347 (1.4)	27 (1.0)	<0.001
Epilepsy (%)	712 (0.5)	138 (0.4)	181 (0.5)	213 (0.5)	161 (0.6)	19 (0.7)	<0.001
Coronary heart disease / myocardial infarction (%)	9463 (6.4)	36 (0.1)	635 (1.7)	3105 (7.3)	5177 (20.5)	510 (19.4)	<0.001
Atrial fibrillation (%)	1263 (0.9)	10 (0.0)	55 (0.1)	283 (0.7)	782 (3.1)	133 (5.1)	<0.001
Hip fracture (%)	277 (0.2)	23 (0.1)	6 (0.0)	22 (0.1)	219 (0.9)	7 (0.3)	<0.001
Spine fracture (%)	451 (0.3)	21 (0.1)	30 (0.1)	80 (0.2)	294 (1.2)	26 (1.0)	<0.001
Moderate or severe liver disease, Liver cirrhosis (%)	1068 (0.7)	75 (0.2)	185 (0.5)	450 (1.1)	271 (1.1)	87 (3.3)	<0.001
Any malignancy, non-metastatic (%)	4865 (3.3)	174 (0.4)	662 (1.7)	1531 (3.6)	1679 (6.7)	819 (31.2)	<0.001
Thromboembolism: prosthetic valve, thrombosis, embolism (%)	20 (0.0)	1 (0.0)	4 (0.0)	6 (0.0)	6 (0.0)	3 (0.1)	<0.001
Pressure Ulcer (%)	230 (0.2)	4 (0.0)	10 (0.0)	43 (0.1)	155 (0.6)	18 (0.7)	<0.001
Heart failure and Fluid overload (%)	2167 (1.5)	51 (0.1)	153 (0.4)	478 (1.1)	1292 (5.1)	193 (7.4)	<0.001
Peripheral vascular disease (%)	1105 (0.8)	15 (0.0)	73 (0.2)	342 (0.8)	558 (2.2)	117 (4.5)	<0.001
**End of Life**							
Metastatic disease	829 (0.6)	23 (0.1)	98 (0.3)	181 (0.4)	241 (1.0)	286 (10.9)	<0.001

Num Numbers were presented as number (%). A complex chronic disease is defined as one that that interfere with / restrict normal function or sufficient to trigger care scare seeking.

### Health services utilization patterns

In terms of health services utilization patterns in 2012, S1 Fig showed the relative healthcare utilization of each cluster using population mean as reference. S2 Fig demonstrated the proportion of healthcare utilization by each segment. The differences in all types of healthcare utilizations between the five segments were statistically significant with p < 0.001. The “Young, healthy” and “Middle age, healthy” segments consist of low-needs subjects. Subjects in the “Young, healthy” segment are young and their inpatient admissions, specialist outpatient visits, emergency department visits, and public primary care clinic visits in 2012 were all lower than population mean as shown in S1 Fig. Similar to the “Young, healthy” segment, subjects in the “Middle age, healthy” segment had overall low healthcare utilization in 2012. Compared to “Young, healthy” segment, however, “Middle age, healthy” segment had higher primary care clinic visits and lower inpatient admissions which correlated to older age and higher prevalence of chronic stable diseases in this group (Table 2). Together, they made up of more than 50% of the population but only consumed 11% of inpatient admissions, 10% of specialist outpatient visits, 18% of emergency department visits, and 24% of public primary care clinic visits as shown in S1 Fig. Overall, “Young healthy” and “Middle age, healthy” segments had low morbidity prevalence (Table 2).

Subjects in “Stable, chronic disease” segment were older, with primary care clinic visits higher than population average, but had low inpatient admissions, specialist outpatient visits, and emergency department visits. For subjects in “Complicated chronic disease” and “Frequent admitters” segments, all types of health services utilization in 2012 were higher than population average. Specifically, “Complicated chronic disease” segment had the highest primary care clinic visits among all segments, with inpatient admissions, specialist outpatient visits, and emergency department visits also higher than population average. Subjects in “Frequent admitters” segment had very high healthcare utilization in 2012, with inpatient admissions, specialist outpatient visits, emergency department visits being the highest among all segments, and primary care clinic visits also higher than population average. The “Frequent admitters” segment had the smallest number of subjects (1.79% of the population) but consumed 69% of inpatient admissions, 77% of specialist outpatient visits, 54% of emergency department visits, and 23% of primary care clinic visits.

### Bivariate analyses of segments versus healthcare utilization and mortality from year 2013 to 2016

We observed similar healthcare utilization pattern for 2013–2016 as were seen in 2012. The “Young, healthy” and “Middle age, healthy” segments had low overall healthcare utilization. The “Stable, chronic disease” segment had moderate primary care clinic visits but continued to have low inpatient admissions, specialist outpatient visits, and emergency department visits. “Complicated chronic disease” segment had high primary care clinic visits, moderate inpatient admissions, specialist outpatient visits, and emergency department visits. “Frequent admitters” segment had very high overall healthcare utilization, especially inpatient admissions, specialist outpatient visits, and emergency department visits. The differences between the five segments are all statistically significant with p < 0.001 for all different types of healthcare utilizations from 2013–2016.

### Multivariate negative binomial regression on healthcare utilization from year 2013 to 2016

We used “Younger healthy” segment as the reference group for other segments. Compared to the “Young, healthy” segment, except for “Middle age, healthy” segment, patients in all other segments had significantly higher rate of inpatient admissions from 2013 to 2016 (p < 0.001) after adjusting for age, gender, ethnicity and healthcare utilization in 2012 (Table 3). Patients in the “Frequent admitters” segment had the highest Incidence Rate Ratio (IRR) (14.87, 95% Confidence Interval (CI): 13.49–16.40) for inpatient admissions from 2013 to 2016 compared to “Young, healthy” segment. Patients in “Complicated chronic disease” segment also had high IRR (4.69, 95% CI 4.49–4.90). Patients in “Middle age, healthy” had slightly lower rate of inpatient admissions from 2013 to 2016 (IRR 0.91, 95% CI 0.88–0.95, p<0.001). All the other segments had significantly higher utilization in specialist outpatient clinics visits and emergency department visits than “Young, healthy” segment from 2013 to 2016 (all p < 0.001) after adjusting for age, gender, ethnicity and healthcare utilization in 2012. Patients in “Frequent admitters” and “Complicated chronic disease” segments had 15.52 times (95% CI: 14.46–16.64) and 2.65 times (95% CI: 2.58–2.73) the specialist outpatient clinics visits for patients in “Young, healthy” segment, respectively. For emergency department visits, patients in the “Frequent admitters” segment had the highest IRR of 11.09 (95% CI: 10.08–12.19) and patients in “Complicated chronic disease” segment had the second highest IRR of 4.83 (95% CI: 4.64–5.04). For primary care utilization, compared to the “Young, healthy” segment, patients in all other segments had significantly higher utilization (p < 0.001) after adjusting for age, gender, ethnicity and healthcare utilization in 2012. Patients in the “Complicated chronic disease” had the highest IRR of 4.57 (95% CI: 4.46–4.67) followed by “Middle age, healthy” with the second highest IRR of 3.87 (95% CI: 3.79–3.94). Pair-wise segment comparisons for “Middle-age healthy” segment, “Stable, chronic disease” segment, “Complicated chronic disease” segment, and “Frequent admitters” segment was done using Chi-square tests. The results showed that there were significant differences between all segment pairs with p < 0.001 (with Bonferroni correction) for all types of healthcare utilizations 2013–2016.

**Table 3:** Multivariate negative binomial regression on hospital utilization and Cox proportional hazards regression on mortality from Year 2013 to 2016.

	**IRR**	**95% Confidence Interval**	**p-value**
**No. of Inpatient Admissions from 2013 to 2016**			
Segment 1: Young, healthy	1.00	*Reference*	
Segment 2: Middle-age, healthy	0.91	(0.88, 0.95)	<0.001
Segment 3: Stable, chronic disease	1.70	(1.63, 1.77)	<0.001
Segment 4: Complicated chronic disease	4.69	(4.49, 4.90)	<0.001
Segment 5: Frequent admitters	14.87	(13.49, 16.40)	<0.001
**No. of Specialist Outpatient Clinic Visits from 2013 to 2016**			
Segment 1: Young, healthy	1.00	*Reference*	
Segment 2: Middle-age, healthy	1.23	(1.20, 1.26)	<0.001
Segment 3: Stable, chronic disease	1.94	(1.89, 1.99)	<0.001
Segment 4: Complicated chronic disease	2.65	(2.58, 2.73)	<0.001
Segment 5: Frequent admitters	15.52	(14.46, 16.65)	<0.001
**No. of Emergency Department Visits from 2013 to 2016**			
Segment 1: Young, healthy	1.00	*Reference*	
Segment 2: Middle-age, healthy	1.22	(1.18, 1.27)	<0.001
Segment 3: Stable, chronic disease	1.92	(1.85, 2.00)	<0.001
Segment 4: Complicated chronic disease	4.83	(4.64, 5.04)	<0.001
Segment 5: Frequent admitters	11.09	(10.08, 12.19)	<0.001
**No. of Primary Care Clinic Visits from 2013 to 2016**			
Segment 1: Young, healthy	1.00	*Reference*	
Segment 2: Middle-age, healthy	2.13	(2.09, 2.17)	<0.001
Segment 3: Stable, chronic disease	3.87	(3.79, 3.94)	<0.001
Segment 4: Complicated chronic disease	4.57	(4.46, 4.67)	<0.001
Segment 5: Frequent admitters	3.53	(3.33, 3.73)	<0.001
	**Hazard Ratio**	**95% Confidence Interval**	**p-value**
**Mortality Rate from 2013 to 2016**			
Segment 1: Young, healthy	1.00	*Reference*	
Segment 2: Middle-age, healthy	1.16	(0.91, 1.50)	0.236
Segment 3: Stable, chronic disease	1.22	(0.94, 1.57)	0.133
Segment 4: Complicated chronic disease	1.95	(1.45, 2.61)	<0.001
Segment 5: Frequent admitters	8.16	(6.17, 10.81)	<0.001

Models are adjusted for age, gender, and ethnicity, past healthcare utilization in 2012. Survival time was used as exposure variable for Negative Binomial Regression.

### Analysis of survival time from year 2013 to 2016

Patients in the “Frequent admitters” performed worst in the 4-year survival analysis (survival rate 74.53%), followed by patients in “Complicated chronic disease” segment with survival rate of 83.79% (Table 3 and S3 Fig). The survival rates for patients in the Segments “Young, healthy”, “Middle age, healthy” and “Stable, chronic disease” were greater than 90% survival rates with “Young, healthy” being the highest (99.77%) followed by “Middle age, healthy” (99.23%) and “Stable, chronic disease” (97.07%). The log-rank test for equality of the five survival curves showed statistically significant differences between the five distributions with p < 0.001. Compared to the “Young, healthy” segment, patients in “Complicated chronic disease” segments and “Frequent admitters” segment had significantly higher rate of mortality from 2013 to 2016 (p < 0.001) whereas patients in “Middle-age, healthy” segment and “Stable, chronic disease” segment did not have significantly different mortality from 2013–2016 after adjusting for age, gender, ethnicity and healthcare utilization in 2012. Patients in “Frequent admitters” segment had the highest Cox Hazard Ratio (8.16, 95% CI: 6.17–10.81).

## Discussion

Using data-driven cluster analysis based on age and healthcare utilization variables, our study identified five, relatively homogeneous patient groups with distinct disease patterns, healthcare utilization and mortality risk. To the best of our knowledge, our study is the first to address a critical gap in literature by demonstrating the validity of our healthcare utilization based approach segmentation on long term follow on healthcare utilization and mortality data. In this regard, we achieved our aim to find the smallest number of health profiles that described the association among the set of observed health utilization patterns.

Naming of clusters is a subjective process and we named our clusters in a way which best represented the disease and utilization patterns. Our findings validate existing literature by confirming a large proportion of healthy population, in addition to a continuum of severity for chronic diseases. It is also worth noting that chronic diseases can be segmented into parsimonious risk groups (three in our study) without the need for extensive classification as in the John Hopkins ACG [12] and 3M CRG [13] systems which adjust risk for patient payments. This is a reasonable first-cut at the policy understanding and planning level and channeling valuable resources to deep-dive into the higher-risk segments to better understand the drivers and risk factors for undesirable clinical outcomes. A recent study based on an experts-driven segmentation framework Senior Segmentation Algorithm [14] identified individuals at increased risk for higher healthcare costs amongst Medicare or Medicaid enrollees [41]. The study demonstrated higher number of healthcare episodes and healthcare cost in the higher-risk tier patients using one-year period follow up data [41]. Another experts-driven segmentation study using CRG system [13] incorporated functional health status data to enhance risk adjustment and showed improved accuracy in cost estimation [42]. In another study on Medicaid enrollees, using COMPLEXedex^TM^ algorithm, an experts-driven, diagnosis-based segmentation method, the 30-day inpatient readmission rates ranged from 6% in the non-chronic segment to 12% in the chronic disease complexity segment and 21% in the organ system failure complexity segment [43]. These recent studies had important implications in effective healthcare resource allocation and the financing of care costs; however, the study populations were restricted to socioeconomically disadvantaged individuals and did not have long-term follow up data that are important for future policy making. The current study addressed these gaps by inclusion of big sample of general patient population and 4 year longitudinal health data with no loss of follow up.

Disease prevention, health education and robust primary care remain the strategies to maintain the health status of the “Young, healthy” and “Middle age, healthy” segments [44]. However, their low healthcare utilization presents a unique challenge because they had little contact with the healthcare institutions. Working with non-healthcare partners such as employers, community-based service providers to design community-based disease education programs and preventive services such as ambulatory screening tests[45] will extend the health system’s reach. Patients in “Stable, chronic disease” had higher primary care utilizations with higher prevalence in stable chronic diseases. They may benefit more from supportive self-management such as home-based self-monitoring tools or telehealth monitoring [27] to promote health empowerment. Patients in “Complicated chronic disease” and “Frequent admitters” segments, on the other hand, require more intensive disease management and multidisciplinary medical and social care coordination. Given the higher prevalence of malignancy, organ failures and high mortality rate in “Frequent admitters” segment, end-of-life care should be one of the priorities for this group of patients. For example, Advance Care Planning (ACP) was found to be associated with lower medical expenditures at the end of life and less distress among patients and their family members[46]. Carefully targeted educational interventions and community programs may encourage such preparations and lead to better care outcome.

Our study addressed some key gaps in recent studies. Vuik et al. previously explored the potential of using healthcare utilization-based clustering analysis for population segmentation [19]. However, the variables used for clustering did not cover emergency department visits, which had important value in understanding patients’ health status and healthcare utilization patterns across the healthcare continuum. Our study addressed this gap by covering utilization data on primary care, inpatient admissions, outpatient visits, and emergency department visits. Zayas et al. also assessed the effectiveness of population segmentation by healthcare utilization patterns but only included elderly and middle-aged adults [28].

An important limitation of our study is that cross-institution health services utilizations were not captured in our current database. Patients may have healthcare utilization beyond SingHealth RHS but we minimized this limitation by only including resident population whose postal codes fall into SingHealth RHS catchment region. Additional studies to validate our cluster analysis derived segments will be required in other populations.

Future research directions should focus on further profiling of specific patient segments. Evidence suggests wider determinants of health, including lifestyle, living environment, emotional, and socio-economic factors, in addition to medical factors[47]. Clustering analysis on a heterogeneous high-utilization segment such as the “Complicated chronic disease” and “Frequent admitters” segments by socio-economic, functional, and behavioral variables could provide further “person-centred” information about the determinants of health and health preferences within the segments so as to enable more tailored and effective interventions. More perspective, longitudinal data are also needed to identify the movement of patients between segments and examine the medical, socio-economic, and behavioral determinates of the movement directions.

## Conclusion

Our data-driven clustering analysis on a general patient population in Singapore identified five patient segments with distinct longitudinal healthcare utilization patterns and mortality risk to provide an evidence-based, quantitative overview of a regional health system’s healthcare needs. This is critical to facilitate the policy makers’ development of population health policy strategies and design of targeted healthcare service packages that meet each segment’s specific needs. Further research is needed to refine the segmentation tool and profile the drivers in the high-utilization segments. More perspective, longitudinal data to examine the potential movement of patients between segments and the various determinants of the movement directions can further facilitate the development of focused health intervention programs.

## Supporting information

S1 Fig Relative healthcare utilization 2012 by segments. (DOCX)

S2 Fig Utilization in year 2012 for each type of health services by segments. (DOCX)

S3 Fig Kaplan-Meier survival estimate by patient segment. (DOCX)
