# Supplementary material for: Assessing the validity of a data driven segmentation approach: A 4 year longitudinal study of healthcare utilization and mortality
Source: PLoS One. 2018 Apr 5;13(4):e0195243. doi: 10.1371/journal.pone.0195243 (PMC5886524; doi:10.1371/journal.pone.0195243)
Supplement: S1 Fig — (DOCX) [file pone.0195243.s001.docx]

S1 Figure. Relative Healthcare Utilization 2012 by Segments

Numbers were presented as percentage difference from population mean
