# Supplementary material for: Assessing the validity of a data driven segmentation approach: A 4 year longitudinal study of healthcare utilization and mortality
Source: PLoS One. 2018 Apr 5;13(4):e0195243. doi: 10.1371/journal.pone.0195243 (PMC5886524; doi:10.1371/journal.pone.0195243)
Supplement: S2 Fig — (DOCX) [file pone.0195243.s002.docx]

S2 Figure. Utilization in year 2012 for each type of Health Services by Segments
