# Supplementary material for: Assessing the validity of a data driven segmentation approach: A 4 year longitudinal study of healthcare utilization and mortality
Source: PLoS One. 2018 Apr 5;13(4):e0195243. doi: 10.1371/journal.pone.0195243 (PMC5886524; doi:10.1371/journal.pone.0195243)
Supplement: S3 Fig — (DOCX) [file pone.0195243.s003.docx]

S3 Figure. Kaplan-Meier Survival Estimate by Patient Segment

| **Days** | **0** | **180** | **360** | **540** | **720** | **900** | **1080** | **1260** | **1440** |
| --- | --- | --- | --- | --- | --- | --- | --- | --- | --- |
| Number at Risk |  |  |  |  |  |  |  |  |  |
| Segment 1: Young, healthy | 41160 | 41146 | 41140 | 41131 | 41124 | 41111 | 41100 | 41093 | 41079 |
| Segment 2: Middle-age, healthy | 38337 | 38308 | 38277 | 38257 | 38224 | 38185 | 38135 | 38088 | 38045 |
| Segment 3: Stable, chronic disease | 40905 | 40800 | 40694 | 40596 | 40493 | 40348 | 40198 | 40044 | 39883 |
| Segment 4: Complicated chronic disease | 22818 | 22427 | 22120 | 21742 | 21374 | 20909 | 20484 | 19997 | 19553 |
| Segment 5: Frequent admitters | 2459 | 2347 | 2232 | 2155 | 2099 | 2034 | 1985 | 1933 | 1897 |
